# Medicinal Plants Traded in Informal Herbal Medicine Markets of the Limpopo Province, South Africa

**DOI:** 10.1155/2019/2609532

**Published:** 2019-04-16

**Authors:** Marula Triumph Rasethe, Sebua Silas Semenya, Alfred Maroyi

**Affiliations:** ^1^Limpopo Department of Economic Development, Environment and Tourism, Private Bag X9484, Polokwane 0700, South Africa; ^2^Technology Transfer Office, Research Administration and Development Department, University of Limpopo, Private Bag X1106, Sovenga 0727, South Africa; ^3^Medicinal Plants and Economic Development (MPED) Research Centre, Department of Botany, University of Fort Hare, Private Bag X1314, Alice 5700, South Africa

## Abstract

Trading of herbal medicines generates economic opportunities for vulnerable groups living in periurban, rural, and marginalized areas. This study was aimed at identifying medicinal plant species traded in the Limpopo province in South Africa, including traded plant parts, conservation statutes of the species, and harvesting methods used to collect the species. Semistructured questionnaire supplemented by field observation was used to collect data from owners of 35 informal herbal medicine markets in the Limpopo province. A total of 150 medicinal plant products representing at least 79 plant species belonging to 45 botanical families, mainly the Fabaceae (11.4%), Asteraceae (7.6%), and Hyacinthaceae (6.3%), were traded in the study area. Roots (50.0%), bulbs (19.0%), and bark (16.0%) were the most frequently sold plant parts. Some of the traded species which include* Alepidea amatymbica, Bowiea volubilis, Brackenridgea zanguebarica, Clivia caulescens*,* Dioscorea sylvatica*,* Elaeodendron transvaalense*, Encephalartos woodii,* Eucomis pallidiflora* subsp.* pole-evansii,* Merwilla plumbea,* Mondia whitei*,* Prunus africana, Siphonochilus aethiopicus, Synaptolepis oliveriana, *and* Warburgia salutaris *are of conservation concern and listed on the South African Red Data List. Findings of this study call for effective law enforcement to curb illegal removal of wild plants especially those species that are at the verge of extinction.

## 1. Introduction

Research by Olsen [2] and Djordjevic [3] estimated that 70% to 80% of the people in developing countries use raw medicinal plants to meet their primary health care needs. This high percentage is attributed to several factors including limited accessibility, availability, and affordability of modern medicines [4, 5]. Generally, the number of African plant species with therapeutic uses is estimated to be close to 6000 [6]. Therefore, it is not surprising that trading of medicinal plant species through informal herbal medicine markets in Africa has significant socioeconomic importance in various countries, as this enable millions of people to generate incomes [7–17]. Quiroz et al. [16] argued that herbal medicines generate economic opportunities for vulnerable groups living in periurban, rural, and marginalized areas especially women and farmers facing decreasing agricultural incomes. Meke et al. [18] argued that 90% of herbal traders in southern and central Malawi derived more than 50% of their households' income from selling medicinal plants. Similarly, over 61 000 kilograms of nonpowdered medicines valued US$344,882 are traded in informal herbal medicine markets of Tanzania per year [19]. In Morocco, annual revenues generated from export of medicinal plants were US$55.9 million in 2015 [20] and US$174, 227,384 in Egypt [21]. According to van Andel et al. [15], approximately 951 tonnes of crude herbal medicines with an estimated total value of US$7.8 million was traded in Ghana's herbal markets in 2010. Findings from all these aforesaid studies show that trading in medicinal plants play an important socioeconomic role in several Africans countries.

Similarly, trading in medicinal plants also serves as a valuable source of income for several households in different provinces of South Africa. Mander et al. [13] argued that the trade in herbal medicines in South Africa is estimated to generate an income value at about R2.9 billion per year, representing about 5.6% of the National Health budget. For example, in KwaZulu, Natal province, between 20000 and 30000 people, mainly woman make a living from trading over 4000 tonnes of medicinal plant materials valued at R60 million per year [9]. Dold and Cocks [10] found that a total of 166 medicinal plant species estimated to be 525 tonnes and valued about R27 million are traded in the Eastern Cape province annually. In the Limpopo province, research by Botha et al. [22] showed that 70 plant species were traded in Sibasa and Thohoyandou in Vhembe district, Giyani, and Malamulele in Mopani district. Moeng [23] found that each medicinal plant trader in the Limpopo province generated more than R5000 per month. There are concerns that the trade in traditional medicines threatens the wild populations of the utilized species as a result of harvesting pressure [8, 9, 13, 17, 24].

The trade in herbal medicines in South Africa is on a scale that is a cause for concern among researchers, conservation organizations, and traditional healers as the harvesting methods employed are unsustainable [9, 13, 17, 23, 25–31]. The harvesting methods employed by medicinal plant gatherers involve uprooting of whole plants, collection of roots, bulbs, removal of the bark, and cutting of stems and leaves. These harvesting methods are aimed at collecting large quantities of medicinal plants including those that are of conservation concern and in some cases illegally collecting plant materials in protected areas and critically endangered ecosystems. Consequently, the population numbers of these targeted medicinal plants are declining rapidly and some of them are now on the verge of extinction leaving their therapeutic potential unfulfilled. The current study was, therefore, aimed at documenting medicinal plants traded in the Limpopo province, including traded plant parts, conservation statutes of the species, and harvesting methods used to collect the species. This information will provide the insight into commercial trade of medicinal plants in the Limpopo province, information on targeted species, the economic value, and possible ecological impacts of the species.

## 2. Research Methods

### 2.1. Study Area and Markets Survey

**Figure 1:**
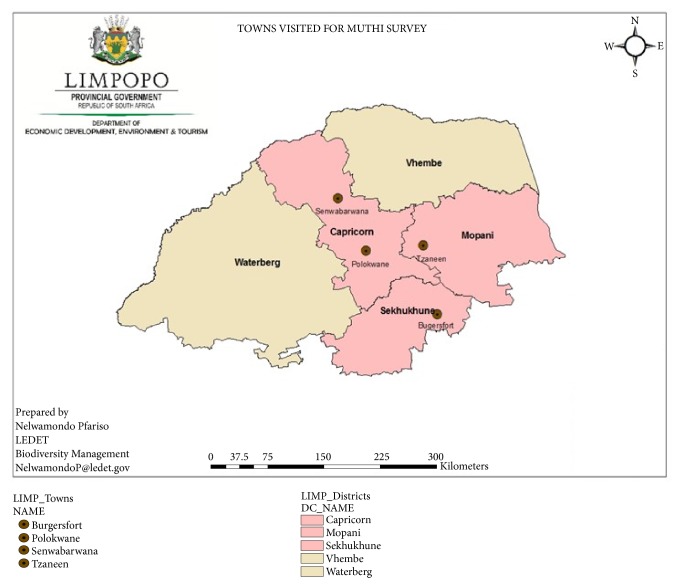
Map of the study area indicating surveyed informal herbal medicine shops and districts.

The present study was conducted in all five districts (Capricorn, Mopani, Sekhukhune, Waterberg and Vhembe) of the Limpopo Province of South Africa (Figure 1). In each district, seven informal herbal medicine shops were sampled, resulting in 35 shops visited in the study area. The shop owners who were directly involved in marketing medicinal plants in these shops were interviewed. The participants were informed about the aim and objectives of the study before being requested to sign the consent form. The researchers adhered to the ethical guidelines outlined by the International Society of Ethnobiology (http://www.ethnobiology.net/what-we-do/core-programs/ise-ethics-program/code-of-ethics/). The ethical clearance to conduct this study was obtained from the Limpopo Department of Economic Development, Environment, and Tourism (LEDET) and the survey was conducted from January 2016 to March 2018. Data was gathered using a semistructured interview, which was supplemented by market observations and field visits to determine harvesting methods and habitats of the traded plant species. The latter activity was conducted together with the participants. Other documented information included sociodemographic profiles of the participants, plant parts used, sources of traded plants, and conservation statutes of the documented species.

### 2.2. Plant Specimen Collection and Data Analysis

Dold and Cocks [10] argued that the use of vernacular names to identify taxa traded in informal herbal medicine markets is unreliable as they vary considerably from place to place and even between traders within the same market. Therefore, to positively identify the plant material traded in the sampled herbal medicine markets, we requested traders to accompany us to the field. In this regard, the traders initially identified the plants using their vernacular names and during field trips the voucher specimens of these species were collected and their identities authenticated at the University of Limpopo's Larry-Leach Herbarium. Botanical names and the plant families of the documented species were confirmed using the ‘The Plant List' created by the Missouri Botanical Gardens and the Royal Botanic Gardens, Kew (http://www.theplantlist.org/).

Information gathered from the interview schedules and field observations was collated and analyzed using Microsoft Excel 2000 and the Statistical Package for the Social Science (SPSS) version 16.0 programme. Descriptive statistics such as percentages frequencies were used. The conservation statutes of traded medicinal plant species were categorized following the IUCN Red List Criteria Version 3.1 (2001). Species can be classified into one of the three categories of threat, that is, Critically Endangered (CR), Endangered (EN), or Vulnerable (VU), or they are placed into Near Threatened (NT), Data Deficient (DD), Extinct (EX), or Extinct in the Wild (EW). If a species does not meet any of these criteria, it is classified as Least Concern (LC). A species classified as LC can additionally be flagged as being of conservation concern either as Rare, Critically Rare, or Declining [32, 33].

## 3. Results and Discussion

### 3.1. Sociodemographic Profiles of Participants

The majority (n=33, 94.2%) of medicinal plants traders interviewed in this study were men, and females constituted 5.7% (n=2). The predominance of men in trading herbal medicines is common in Malawi [18], South Africa [22], and Tanzania [19]. However, Mander et al. [13] found that the majority of medicinal plant traders in the Gauteng, Mpumalanga and KwaZulu-Natal provinces of South Africa were women. Ndawonde et al. [34] found that 77% of the 63 plant traders interviewed in KwaZulu-Natal province were women. Results of the current study revealed that the male participants were the custodians of the species traded in the province and the associated indigenous knowledge, innovations, and practices. These findings corroborate the observation made by Cunningham [7] that the commercially sold medicinal plants are an important feature of the cultural, medicinal, economical, and ecological component of every city in the world.

Close to three quarters of the participants (n = 24, 68.5%) were between 31 and 40 years and 20% (n = 7) were between 21 and 30 years while 11.4% (n = 4) were between 41 and 50 years. Therefore, increasing trade in the medicinal plants is expected in the Limpopo province in the future as the majority of the participants were within the very active age group. More than half of the participants (n = 22, 62.8%) were educated up to secondary education, while 22.8% (n = 8) and 14.2% (n = 5) had attained tertiary and primary education, respectively. The importance of medicinal plants and the need to trade them in the Limpopo province were ubiquitously perceived, with all participants claiming to generate adequate profit to meet their basic livelihood needs and being optimistic about the future of the medicinal plants trade in the province. More than three quarters of the participants (n = 27, 77.1%) earned monthly incomes of between R3000 and R4000.00. The rest of the participants earned monthly incomes of less than R3000 (n = 5, 14.2%) or more than R5 000 (n = 3, 8.5%). The findings of this study emphasize the contribution of herbal medicines trade towards participants' livelihood needs, source of primary health care products, and cultural heritage corroborating research by Mander et al. [13] who argued that trade in herbal medicines in South Africa is a large and growing industry which is important to the national economy.

### 3.2. Diversity of Traded Medicinal Plants

A total of 150 medicinal plant products representing at least 79 plant species were recorded in the surveyed informal herbal medicine shops in the Limpopo province (Table 1). A total of 79 species belonging to 45 botanical families, mainly the Fabaceae (n = 9 spp., 11.4%), Asteraceae (n = 6 spp., 7.6%), Hyacinthaceae (n = 5 spp., 6.3%), Amaryllidaceae (n = 4 spp., 5.1%), Celastraceae, Ebenaceae, and Gentianaceae (n = 3 spp., 3.8% each) were positively identified and, therefore, further analyses were based on these species. The rest of the medicinal plant products were excluded in the current study because they were not positively identified due to lack of diagnostic features such as leaves and fruiting material such as flowers and fruits. Previous studies showed that plant species belonging to Fabaceae (13.0%), Apocynaceae (5.7%), Phyllanthaceae (4.9%), and Rubiaceae (4.1%) were the most traded species in Malawi [18], while Fabaceae (7.4%), Asteraceae (6.7%), and Euphorbiaceae (5.5%) were the most traded taxa in South Africa [27] while Asteraceae and Fabaceae (10.6% each), Euphorbiaceae (8.5%) and Cucurbitaceae (6.4%) were the most traded taxa in Botswana [14]. Plant families Apocynaceae, Asteraceae, Cucurbitaceae, Euphorbiaceae, Fabaceae, Phyllanthaceae, and Rubiaceae have the highest number of traded species as herbal medicines probably because these are large families characterized by several species, at least 989 species each (http://www.theplantlist.org/).

**Figure 2:**
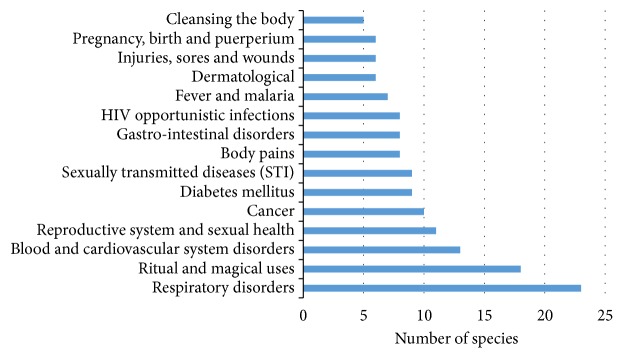
Medicinal categories of traded medicinal plants in the Limpopo province following the Economic Botany Data Collection Standard [1].

Analysis of traded plant species showed that herbs and trees (n = 34, 43% each) and shrubs (n = 11, 13.9%) were the most traded growth forms. Previous research in the Limpopo province carried out by Botha et al. [22] showed that trees, shrubs, herbs, climbers, and geophytes were the most traded growth forms in the province. More than half of the traded species (n = 67, 84.8%) were prescribed for more than one ailment and just 15.1% (n = 12) were sold as herbal medicine for a single ailment (Table 1). Previous studies conducted in the Limpopo [23, 35], Kwa-Zulu Natal [34], and the Northern Cape [36] provinces in South Africa also found that informal herbal medicine traders mainly sold species with multiple medicinal applications. The medicinal applications of the traded species were classified into 15 major medical categories following the Economic Botany Data Collection Standard [1] with some changes proposed by Macía et al. [37] and Gruca et al. [38]. These categories included respiratory disorders, ritual and magical uses, blood and cardiovascular system disorders, reproductive system and sexual health disorders, cancer, diabetes, sexually transmitted infections (STI), body pains, gastrointestinal system disorders, human immunodeficiency virus (HIV) opportunistic infections, fever and malaria, dermatological problems, injuries, sores and wounds, pregnancy, birth and puerperium, and cleansing of the body (Figure 2). The species traded in the Limpopo province are used as herbal medicine against several diseases categorized by Stats SA [39] as the top killer diseases in South Africa in 2016 which include tuberculosis, heart diseases, HIV diseases, influenza, diabetes mellitus, and other viral diseases. Over the years, there have been numerous studies that have validated the traditional uses of some of the traded medicinal plants against the top killer diseases.

### 3.3. Highly Traded Species

The most frequently traded plant species, recorded in 28.6% of the informal herbal medicine shops, included the following (in ascending order of importance):* Osyris lanceolata* Hochst. and Steud.,* Pleurostylia capensis* (Turcz.) Loes.,* Sclerochiton ilicifolius* A. Meeuse,* Hypoxis obtusa* Burch. ex Ker Gawl.,* Bowiea volubilis* Harv. ex Hook.f.,* Callilepsis laureola* DC.,* Clivia caulescens* R.A. Dyer,* Dioscorea sylvatica* Eckl., Dioscorea dregeana (Kunth) T Durand and Schinz,* Brackenridgea zanguebarica* Oliv.,* Securidaca longepedunculata* Fresen.,* Zanthoxylum capense* (Thunb.) Harv.,* Alepidea amatymbica* Eckl. and Zeyh.,* Drimia elata *Jacq.,* Enicostema axillare* (Lam) A Raynal subsp.* axillare*,* Eucomis autumnalis* (Mill) Chitt.,* Hypoxis hemerocallidea *Fisch, C A Mey and Avé-Lall,* Monsonia angustifolia *Sond.,* Siphonochilus aethiopicus* (Schweinf) B L Burtt, and* Warburgia salutaris *(G Bertol) Chiov (Tables 1 and 2). More than half of the participants indicated that the following species were in high demand but not readily available in the wild or rare or their populations declining (Table 2):* Bowiea volubilis*,* Clivia caulescens*,* Dioscorea sylvatica*, Dioscorea dregeana,* Brackenridgea zanguebarica*,* Alepidea amatymbica*,* Eucomis autumnalis*,* Siphonochilus aethiopicus, *and* Warburgia salutaris*. With the exception of* Dioscorea dregeana *and* Eucomis autumnalis* these species are listed on the South African Red Data List as threatened plant species (Table 1), with* Brackenridgea zanguebarica *and* Siphonochilus aethiopicus *listed as Critically Endangered,* Alepidea amatymbica* and* Warburgia salutaris* listed as Endangered, and* Bowiea volubilis* and* Dioscorea sylvatica* listed as Vulnerable while* Clivia caulescens* is listed as Near Threatened [40]. Six other plant species that are traded in the study area but not categorized as high in demand by the participants which are of conservation concern in South Africa and listed on the South African Red Data List include the following (Table 1):* Encephalartos woodii* Sander which is listed as Extinct in the Wild;* Mondia whitei* (Hook.f.) Skeels is listed as Endangered;* Prunus africana* (Hook.f.) Kalkman is listed as Vulnerable while* Elaeodendron transvaalense* (Burtt) R H Archer,* Merwilla plumbea *(Lindl.) Speta,* Eucomis pallidiflora* Baker subsp.* pole-evansii* (N E Br) Reyneke ex J C Manning, and* Synaptolepis oliveriana* Gilg are listed as Near Threatened [40].* Interviews with participants revealed that Alepidea amatymbica, Bowiea volubilis, Brackenridgea zanguebarica, Clivia caulescens, Dioscorea sylvatica*, Dioscorea dregeana,* Eucomis autumnalis, Sclerochiton ilicifolius, Siphonochilus aethiopicus, *and* Warburgia salutaris *which were regarded as popular and harvested from the wild were becoming locally extinct and these species fetched high prices (Table 2).* All these 14 species that are traded in the Limpopo province but listed* on the South African Red Data List are in general overcollected as herbal medicines and extracted at unsustainable rate throughout their distributional ranges [40].

About three quarters of the participants (n = 26, 74.2%) did not have plant collecting permits as required by law in South Africa [41]. Only a quarter of the participants (n = 9, 25.7%) were in possession of a general plant collecting permit allowing them to collect any medicinal plants in the wild, without stating the quotas of materials to be harvested, use of sustainable harvesting techniques, and restrictions on the collection of threatened and protected plants. According to Retief et al. [42] a Threatened or Protected Species (TOPS) permit in terms of the National Environmental Management: Biodiversity Act (NEM:BA) of 2004 is required to collect and possess the following species which were traded by the participants and listed on the South African Red Data List:* Alepidea amatymbica, Bowiea volubilis, Brackenridgea zanguebarica, Clivia caulescens*,* Dioscorea sylvatica*,* Elaeodendron transvaalense*, Encephalartos woodii,* Eucomis pallidiflora* subsp.* pole-evansii,* Merwilla plumbea,* Mondia whitei*,* Prunus africana, Siphonochilus aethiopicus, Synaptolepis oliveriana, *and* Warburgia salutaris. *None of the participants had a TOPS permit, therefore, these species were being illegally harvested by the participants. Findings of this study call for effective law enforcement to curb illegal removal of wild plants especially those species that are at the verge of extinction.

Interviews with participants revealed that common key factors that were considered in determining the price of the traded species included demand and availability of the species and also whether the plant material being sold is raw material or partially processed (Table 2). Prices of traded species ranged from ZAR8.50 (USD0.65) to ZAR30.00 (USD2.30) (Table 2). Plant species which were sold for more than ZAR26.00 (USD2.00) included the following (in ascending order of importance):* Brackenridgea zanguebarica*, Dioscorea dregeana,* Dioscorea sylvatica*,* Eucomis autumnalis, Warburgia salutaris, Zanthoxylum capense*, and* Alepidea amatymbica* (Table 2). Prices recorded in the Limpopo province were lower than prices recorded by Dold and Cocks [10] in the Eastern Cape province for* Alepidea amatymbica*,* Bowiea volubilis*, and* Dioscorea sylvatica* with prices ranging from ZAR14.90 (USD1.90) to ZAR82.40 (USD10.30). Mander et al. [13] argued that there is increasing harvesting pressure on traditional supply areas leading to a growing shortage in supply of popular medicinal plant species which are sustaining livelihoods and providing important health care services to local communities.

### 3.4. Traded Plant Parts

**Figure 3:**
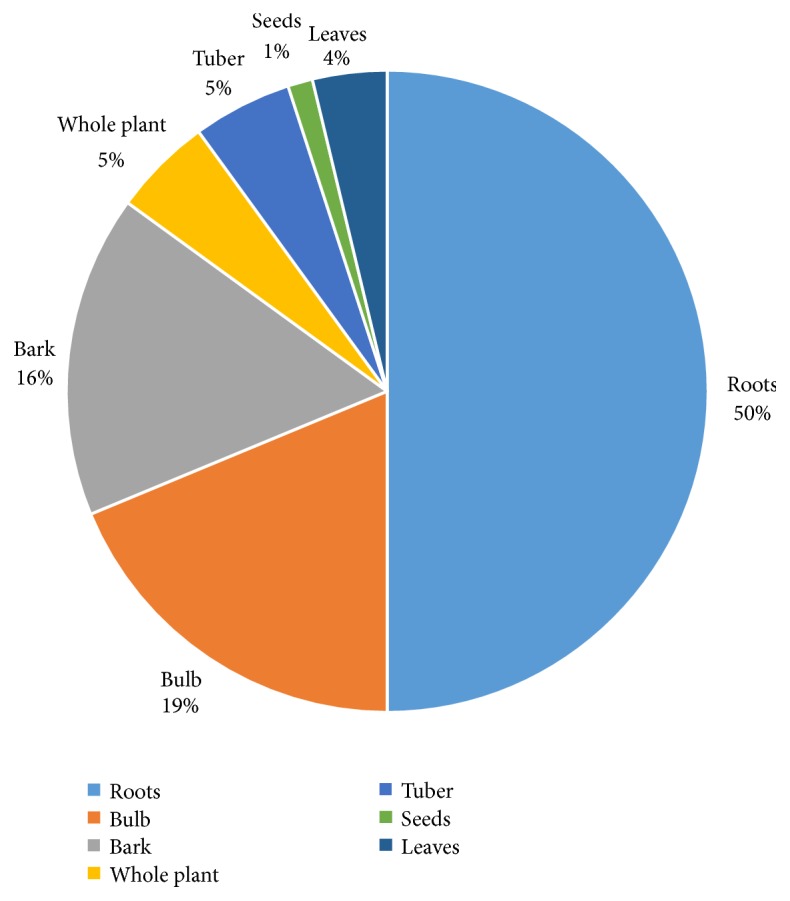
Medicinal plant parts traded in the Limpopo province.

The plant parts traded as herbal medicines in the Limpopo provinces were the bark, bulbs, leaves, roots, seeds, tubers, and whole plant. The roots were the most frequently traded (50.0%), followed by bulbs (19.0%), bark (16.0%), tubers and whole plants (5.0% each), leaves (4.0%), and seeds (1.0%) (Figure 3). The bulbs, tubers, and whole plant were mostly from geophytes and herbaceous plant species (Table 1). However, harvesting of roots of herbaceous plants for medicinal purposes, bark, bulbs, and whole plant is not sustainable as it threatens the survival of the plant species used as herbal medicines. It is well recognized by conservationists that medicinal plants primarily valued for their bark, bulbs, roots, stems, and tubers and as whole plants are often overexploited and threatened [24].

## 4. Conclusion

Medicinal plants are globally valuable sources of pharmaceutical drugs and other health products, but they are disappearing at an alarming rate [24]. Several plant species used as herbal medicines in the Limpopo province are threatened with extinction from overharvesting due to popularity of the species in the herbal medicine markets. Although this threat has been known for decades, the accelerated loss of species and habitat destruction in the province has increased the risk of extinction of medicinal plants in the country. The illegal acquisition of some plant species especially those listed on the South African Red Data List from wild populations is the principle threat to their persistence. There is need, therefore, to educate local communities on the contemporary environmental legislation, at the same time emphasizing the need to retain traditional knowledge on medicinal plant utilization in the province.

## Figures and Tables

**Table 1:** List of plants recorded in informal herbal medicine markets in the Limpopo province, South Africa.

Family	Scientific name	Habit	Conservation status	Part used	Medicinal uses	Frequency (%)
Acanthaceae	*Sclerochiton ilicifolius* A. Meeuse	Tree	LC	Roots	Hypertension and malaria	28.6

Amaryllidaceae	*Clivia caulescens* R.A. Dyer	Herb	NT	Roots	Police evasion	54.3

Amaryllidaceae	*Boophone disticha* (L.f.) Herb.	Herb	LC	Bulb	Cancer, diabetes mellitus and kidney problem	25.7

Amaryllidaceae	*Ammocharis coranica* (Ker Gawl.) Herb.	Herb	LC	Bulb	Hypertension and blood cancer	25.7

Amaryllidaceae	*Clivia miniata* var. citrina	Herb	DDT	Bulb	Human immunodeficiency virus (HIV), arthritis, skin disorder and tuberculosis	22.9

Anacampserotaceae	*Talinum caffrum* (Thunb.) Eckl. & Zeyh	Herb	LC	Roots	Eye disorder	14.3

Anacampserotaceae	*Talinum crispatulum* Dinter	Herb	LC	Roots	Kidney and womb problem	25.7

Anacardiaceae	*Protorhus longifolia* (Bernh.) Engl	Tree	LC	Bark	Tooth decay and bad breath, laxative and poison/kill people	22.9

Anacardiaceae	*Lannea schweinfurthii* (Engl) Engl var. stuhlmannii (Engl)	Tree	LC	Roots	Cause memory loss and increase milk production in pregnant woman	25.7

Annonaceae	*Annona senegalensis *Pers.	Tree	LC	Roots	Chlamydia and malaria	8.6

Apiaceae	*Alepidea amatymbica* Eckl. & Zeyh.	Herb	EN	Roots and whole plant	To attract customers and protection from theft, flu and colds and diabetes	100.0

Apocynaceae	*Acokanthera rotundata* (Codd) Kupicha	Tree	LC	Bark	Headache and sinusitis	11.4

Apocynaceae	*Mondia whitei* (Hook.f.) Skeels	Herb	EN	Roots and fruits	Stamina and diabetes	5.7

Araceae	*Stylochaeton natalensis* Schott	Herb	LC	Roots	Waist pain in men	25.7

Araceae	*Zantedeschia aethiopica* (L.) Spreng.	Herb	LC	Roots	Chlamydia and measles	5.7

Asparagaceae	*Asparagus aethiopicus* L.	Herb	LC	Roots	Attract customers and skin protection for people living with albinism	22.9

Asteraceae	*Callilepis salicifolia* Oliv.	Herb	LC	Roots	Flu, cough and stomach ache	2.9

Asteraceae	*Callilepsis laureola* DC.	Herb	LC	Tuber	Tuberculosis and asthma	54.3

Asteraceae	*Dicoma anomala* Sond.	Herb	LC	Tuber	Tuberculosis and flu	8.6

Asteraceae	*Helichrysum cymosum* (L.) D. Don subsp. *calvum* Hilliard	Shrub	LC	Whole plant	Asthma, call ancestors, cough and tuberculosis	5.7

Asteraceae	*Senecio gregatus* Hilliard	Shrub	LC	Leaves	Body cleansing	22.9

Asteraceae	*Senecio serratuloides* DC.	Shrub	LC	Leaves	Body cleansing and HIV symptoms	22.9

Brassicaceae	*Capparis sepiaria* L. var. subglabra (Oliv) DeWolf	Shrub	LC	Roots	Protection from lightning and nose bleed	2.9

Canellaceae	*Warburgia salutaris *(G.Bertol.) Chiov.	Tree	EN	Bark	Asthma, blood disorders, impotency, skin disorders, sores and tuberculosis	100.0

Capparaceae	*Capparis tomentosa* Lam.	Shrub	LC	Bark	Home protection	11.4

Caryophyllacea	*Dianthus basuticus* Burtt	Herb	LC	Roots	Win court cases	22.9

Celastraceae	*Catha edulis* (Vahl) Forssk ex Endl	Tree	LC	Leaves	Energy booster and stamina	14.3

Celastraceae	*Elaeodendron transvaalense* (Burtt) R H Archer	Tree	NT	Bark	Tuberculosis and sexually transmitted infections (STI)	2.9

Celastraceae	*Pleurostylia capensis* (Turcz.) Loes.	Tree	LC	Roots	Eyes disorders and mental illnesses	28.6

Clusiaceae	*Garcinia gerrardii* Harv. ex Sim	Tree	LC	Roots	Lack of appetite	20.0

Combretaceae	*Combretum molle* R.Br. ex G.Don	Tree	LC	Roots	Skin disorders and tuberculosis	8.6

Commelinaceae	*Commelina subulata* Roth	Herb	LC	Seeds	Cause memory loss	5.7

Dioscoreaceae	*Dioscorea sylvatica* Eckl.	Herb	VU	Bulb	Foot disorder and body pains	54.3

Dioscoreaceae	*Dioscorea dregeana* (Kunth) T Durand & Schinz	Herb	LC	Tuber	Foot disorder, body pains, malaria and to oppress opponents	54.3

Dipsacaceae	*Scabiosa columbaria* L.	Herb	LC	Roots	Assist people to stop drinking alcohol, quench thirst and oral infections	20.0

Ebenaceae	*Diospyros lycioides* Desf subsp *sericea* (Bernh) De Winter	Tree	LC	Roots	Cancer, chest pains and STI	11.4

Ebenaceae	*Euclea crispa* (Thunb.) Gürke subsp. crispa	Tree	LC	Roots	Stomach ailments	22.9

Ebenaceae	*Diospyros galpinii* (Hiern) De Winter	Shrub	LC	Roots	Body cleansing and laxative	2.9

Fabaceae	*Vigna frutescens* A Rich subsp *frutescens* var. *frutescens*	Herb	LC	Roots	Stomach ailments and diarrhoea	20.0

Fabaceae	*Pterocarpus angolensis* DC.	Tree	LC	Bark	Used to cause harm/death	22.9

Fabaceae	*Erythrina lysistemon* Hutch.	Tree	LC	Bark	Cancer, cough, malaria, tuberculosis and skin rash	5.7

Fabaceae	*Peltophorum africanum* Sond.	Tree	LC	Bark	Cleanse body, treat bad luck and HIV symptoms	20.0

Fabaceae	*Mundulea sericea *(Willd.) A. Chev.	Tree	LC	Bark	Tuberculosis and menstrual disorders	20.0

Fabaceae	*Albizia adianthifolia *(Shumach.) W. Wight	Tree	LC	Bark	Mental illnesses, sores and malaria	11.4

Fabaceae	*Elephantorrhiza elephantina* (Burch.) Skeels	Shrub	LC	Roots	Blood disorders, diarrhoea, HIV symptoms and purgative	17.1

Gentianaceae	*Enicostema axillare* (Lam.) A.Raynal subsp. *Axillare*	Herb	LC	Whole plant	Diabetes	100.0

Geraniaceae	*Pelargonium capitatum* (L.) L'Hér.	Herb	LC	Roots	Menstrual pains and labour pains	17.1

Geraniaceae	*Monsonia angustifolia *Sond.	Herb	LC	Whole plant	Diabetes, hypertension, body cleansing, impotency and increase appetite	100.0

Hyacinthaceae	*Drimia elata *Jacq.	Herb	DDT	Bulb	Blood related ailments, periods pains and womb problem	100.0

Hyacinthaceae	*Merwilla plumbea *(Lindl.) Speta	Shrub	NT	Bulb	Body cleansing, skin rash in babies and promote vomiting of impure milk in babies	14.3

Hyacinthaceae	*Urginea sanguinea* Shinz	Herb	LC	Bulb	Hypertension, diabetes, blood clotting, body pains and STI	20.0

Hyacinthaceae	*Eucomis autumnalis* (Mill.) Chitt	Herb	LC	Bulb	Body pains, hypertension and STI	100.0

Hyacinthaceae	*Eucomis pallidiflora* Baker subsp *pole-evansii* (N E Br) Reyneke ex J C Manning	Herb	NT	Bulb	Body pains, hypertension, malaria, STI and tuberculosis	17.1

Hypoxidaceae	*Hypoxis obtusa* Burch. ex Ker Gawl.	Herb	LC	Bulb	Blood disorders, diabetes, hypertension and infertility	34.3

Hypoxidaceae	*Hypoxis hemerocallidea *Fisch, C A Mey & Avé-Lall	Herb	LC	Tuber	Cancer, diabetes, energy booster, hypertension, tuberculosis, infertility and HIV	100.0

Icacinaceae	*Pyrenacantha grandiflora* Baill.	Shrub	LC	Roots	Eye disorders and body pains	20.0

Kirkiaceae	*Kirkia wilmsii* Engl	Tree	LC	Bulb	Arthritis, diabetes, hypertension and quench thirst	11.4

Liliaceae	*Bowiea volubilis* Harv. ex Hook.f.	Herb	VU	Bulb	Blood disorder, cancer and good luck	54.3

Malvaceae	*Dombeya rotundifolia* Hochst.	Tree	LC	Roots	Diarrhoea and stomach ailments	8.6

Malvaceae	*Adansonia digitata *L.	Tree	LC	Bark	Stamina, infertility, impotency and respiratory infections	14.3

Meliaceae	*Ekebergia capensis* Sparrm.	Tree	LC	Roots	Blood cancer and STI	11.4

Meliaceae	*Trichilia emetica *Vahl subsp. Emetic	Tree	LC	Roots	Body cleansing	5.7

Molluginaceae	*Psammotropha marginata* (Thunb.) Druce	Herb	DDT	Roots	Eyes disorders and body pains	17.1

Moraceae	*Ficus ingens *(Miq.) Miq.	Tree	LC	Bark	Sores and stomach disorders	14.3

Myrsinaceae	*Rapanea melanophloeos* (L.) Mez	Tree	LC	Roots	Cancer, wounds and womb problem	8.6

Olacaceae	*Ximenia caffra* Sond var. *caffra*	Tree	LC	Roots	Stomach disorders	17.1

Oleaceae	*Olea europaea* L subsp. *africana* (Mill) P S Green	Tree	LC	Roots	Facilitate birth, cough and tuberculosis	8.6

Passifloraceae	*Adenia spinosa* Burtt Davy	Shrub	LC	Bulb	Bath a baby to promote weight gain	14.3

Polygalaceae	*Securidaca longepedunculata* Fresen.	Tree	LC	Roots	Cough, flu, improve men's fertility and impotency	57.1

Rhamnaceae	*Ziziphus mucronata* Willd. subsp. *mucronata*	Tree	LC	Roots	Cough, STI, sores, tuberculosis and wounds	14.3

Rosaceae	*Prunus africana* (Hook.f.) Kalkman	Tree	VU	Bark	Colds, cough, flu, HIV, stomach complaints and tuberculosis	11.4

Rutaceae	*Zanthoxylum capense* (Thunb.) Harv.	Tree	LC	Roots	Asthma, colds, cough, fix bad situations, flu, sores and tuberculosis	62.9

Rutaceae	*Brackenridgea zanguebarica* Oliv.	Tree	CR	Roots	Reverse bad luck, protect household and protection from evil spirits	57.1

Santalaceae	*Osyris lanceolata* Hochst. & Steud.	Tree	LC	Roots	Attract a woman/man, cause harm/death to people, strengthen a household, reproductive problems and malaria	28.6

Thymelaeacea	*Lasiosiphon caffer* Meisn.	Herb	LC	Roots	Asthma, colds, cough and tuberculosis	14.3

Thymelaeaceae	*Synaptolepis oliveriana* Gilg	Shrub	NT	Roots	Good luck	14.3

Vitaceae	*Rhoicissus tomentosa* (Lam) Wild & R B Drummund	Herb	LC	Roots	Cancer, cough and tuberculosis	17.1

Zamiaceae	*Encephalartos woodii *Sander	Tree	EW	Bulb	Protection against harm	2.9

Zingiberaceae	*Siphonochilus aethiopicus *(Schweinf.) B.L.Burtt	Herb	CR	Bulb	Asthma, colds, cough, body pains, flu, HIV symptoms and good luck	100.0

**Table 2:** The most frequently traded medicinal plants in the Limpopo province.

Species name	Availability in herbal medicine shops during survey	Demand	Availability in the wild	Price range ZAR (USD)/kg*∗*
*Alepidea amatymbica*	Yes (n = 15, 42.9%)	High (n = 35, 100.0%)	Not available (n = 22, 62.9%)	30.00 (2.30)
	Out of stock (n = 20, 57.1%)		Rare (n = 8, 22.9%)	

*Enicostema axillare*	Yes (n = 35, 100.0%)	High (n = 35, 100.0%)	Abundant (n = 35, 100.0%)	15.00 (1.15)

*Eucomis autumnalis*	Yes (n = 29, 82.9%)	High (n = 35, 100.0%)	Declining (n = 22, 62.9%)	27.50 (2.12)
	Out of stock (n = 6, 17.1%)		Rare (n = 9, 25.7%)	
			Not available (n = 1, 2.9%)	

*Hypoxis hemerocallidea*	Yes (n = 21, 60.0%)	High (n = 35, 100.0%)	Abundant (n = 33, 94.3%)	15.00 (1.15)
	Out of stock (n = 14, 40.0%)		Declining (n = 2, 5.7%)	

*Monsonia angustifolia*	Yes (n = 30, 85.7%)	High (n = 35, 100.0%)	Abundant (n = 30, 85.7%)	22.50 (1.73)
	Out of stock (n = 5, 14.3%)		Declining (n = 5, 14.3%)	

*Drimia elata*	Yes (n = 22, 62.9%)	High (n = 35, 100.0%)	Abundant (n = 35, 100.0%)	12.50 (0.96)
	Out of stock (n = 13, 37.1%)			

*Siphonochilus aethiopicus*	Yes (n = 19, 54.3%)	High (n = 35, 100.0%)	Not available (n = 32, 91.4%)	8.50 (0.65)
	Out of stock (n = 16, 45.7%)		Rare (n = 3, 8.6%)	

*Warburgia salutaris*	Yes (n = 31, 88.6%)	High (n = 35, 100.0%)	Declining (n = 29, 82.9%)	27.50 (2.12)
	Out of stock (n = 4, 11.4%)		Rare (n = 6, 17.1%)	

*Zanthoxylum capense*	Yes (n = 11, 31.4%)	High (n = 22, 62.9%)	Abundant (n = 22, 62.9%)	27.50 (2.12)
	Out of stock (n = 11, 31.4%)			

*Securidaca longepedunculata*	Yes (n = 9, 25.7%)	High (n = 20, 57.1%)	Abundant (n = 19, 54.3%)	22.50 (1.73)
	Out of stock (n = 11, 31.4%)		Declining (n = 1, 2.9%)	

*Brackenridgea zanguebarica*	Yes (n = 7, 20.0%)	Moderate (n = 13, 37.1%)	Abundant (n = 2, 5.7%)	27.50 (2.12)
	Out of stock (n = 13, 37.1%)	High (n = 7, 20.0%)	Not available (n = 18, 51.4%)	

*Callilepsis laureola*	Yes (n = 15, 42.9%)	Low (n = 18, 51.4%)	Abundant (n = 18, 51.4%)	12.50 (0.96)
	Out of stock (n = 4, 11.4%)	Moderate (n = 1, 2.9%)		

*Bowiea volubilis*	Yes (n = 9, 25.7%)	High (n = 16, 45.7%)	Rare (n = 14, 40.0%)	12.50 (0.96)
	Out of stock (n = 10, 26.6%)	Moderate (n = 3, 8.6%)	Not available (n = 5, 14.3%)	

*Dioscorea dregeana*	Yes (n = 5, 14.3%)	High (n = 11, 31.4%)	Declining (n = 17, 48.6%)	27.50 (2.12)
	Out of stock (n = 14, 40.0%)	Moderate (n = 6, 17.1%)	Rare (n = 2, 5.7%)	
		Low (n = 2, 5.7%)		

*Dioscorea sylvatica*	Yes (n = 6, 17.1%)	High (n = 19, 54.3%)	Rare (n = 11, 31.4%)	27.50 (2.12)
	Out of stock (n = 13, 37.1%)		Not availableE (n = 8, 22.9%)	

*Clivia caulescens*	Yes (n = 17, 48.6%)	High (n = 9, 25.7%)	Declining (n = 16, 45.7%)	12.50 (0.96)
	Out of stock (n = 2, 5.7%)	Moderate (n = 6, 17.1%)	Rare (n = 2, 5.7%)	
		Low (n = 4, 11.4%)	Abundant (n = 1, 2.9%)	

*Croton gratissimus*	Yes (n = 15, 42.9%)	Low (n = 15, 42.9%)	Abundant (n= 15, 42.9%)	12.50 (0.96)

*Hypoxis obtusa*	Yes (n = 11, 31.4%)	High (n = 9, 25.7%)	Abundant (n = 9, 25.7%)	12.50 (0.96)
	Out of stock (n = 2, 5.7%)	Moderate (n = 3, 8.6%)	Declining (n = 4, 11.4%)	
		Low (n = 1, 2.9%)		

*Osyris lanceolata*	Yes (n = 7, 20.0%)	High (n = 1, 2.9%)	Abundant (n = 7, 20.0%)	12.50 (0.96)
	Out of stock (n = 3, 8.6%)		Declining (n = 3, 8.6%)	

*Pleurostylia capensis*	Yes (n = 1, 2.9%)	High (n = 10, 28.6%)	Abundant (n = 10, 28.6%)	22.50 (1.73)
	Out of stock (n = 9, 25.7%)			

*Sclerochiton ilicifolius *	Yes (n = 8, 22.9%)	High (n = 10, 28.6%)	Declining (n = 7, 20.0%)	22.50 (1.73)
	Out of stock (n = 2, 5.7%)		Rare (n = 3, 8.6%)	

**∗**An average exchange rate for 2018 of USD1 = ZAR13.00 was used.

## Data Availability

The data used to support the findings of this study are included within the article.
